# Supplementary material for: Impact of prescribed fire on soil microbial communities in a Southern Appalachian Forest clear-cut
Source: Front Microbiol. 2024 Apr 25;15:1322151. doi: 10.3389/fmicb.2024.1322151 (PMC11090169; doi:10.3389/fmicb.2024.1322151)
Supplement: Supplementary file 1 [file Data_Sheet_1.pdf]

Supplementary Material

1 Supplementary Figures and Tables

1.1 Supplementary Figures

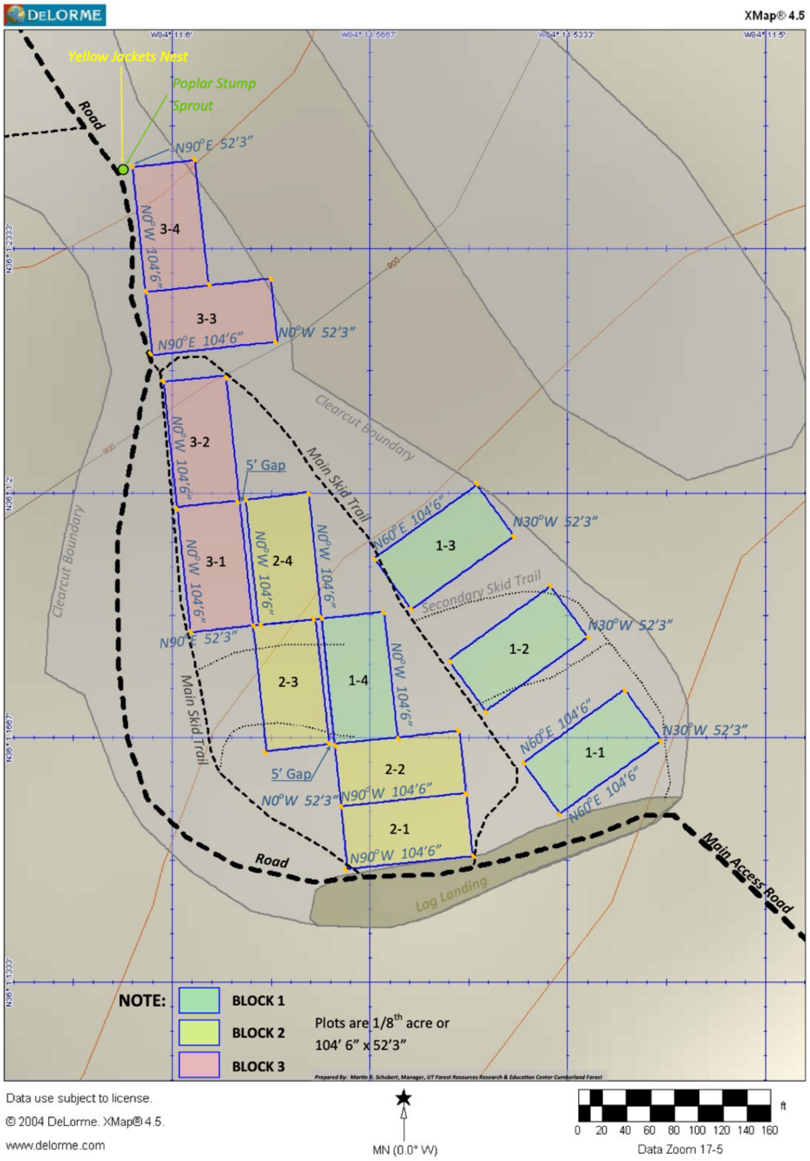

Figure S 1

Detailed Site map for FRREC Burn Site showing plot arrangement and size details. Plots 1-1, 2-3 and 3-2 had additional hardwood slash, Plots 1-2, 2-1, and 3-1 used pine slash, Plots 1-3, 2-4, and 3-4 had no additional slash fuel while plots 1-4, 2-2 and 3-3 were control plots.

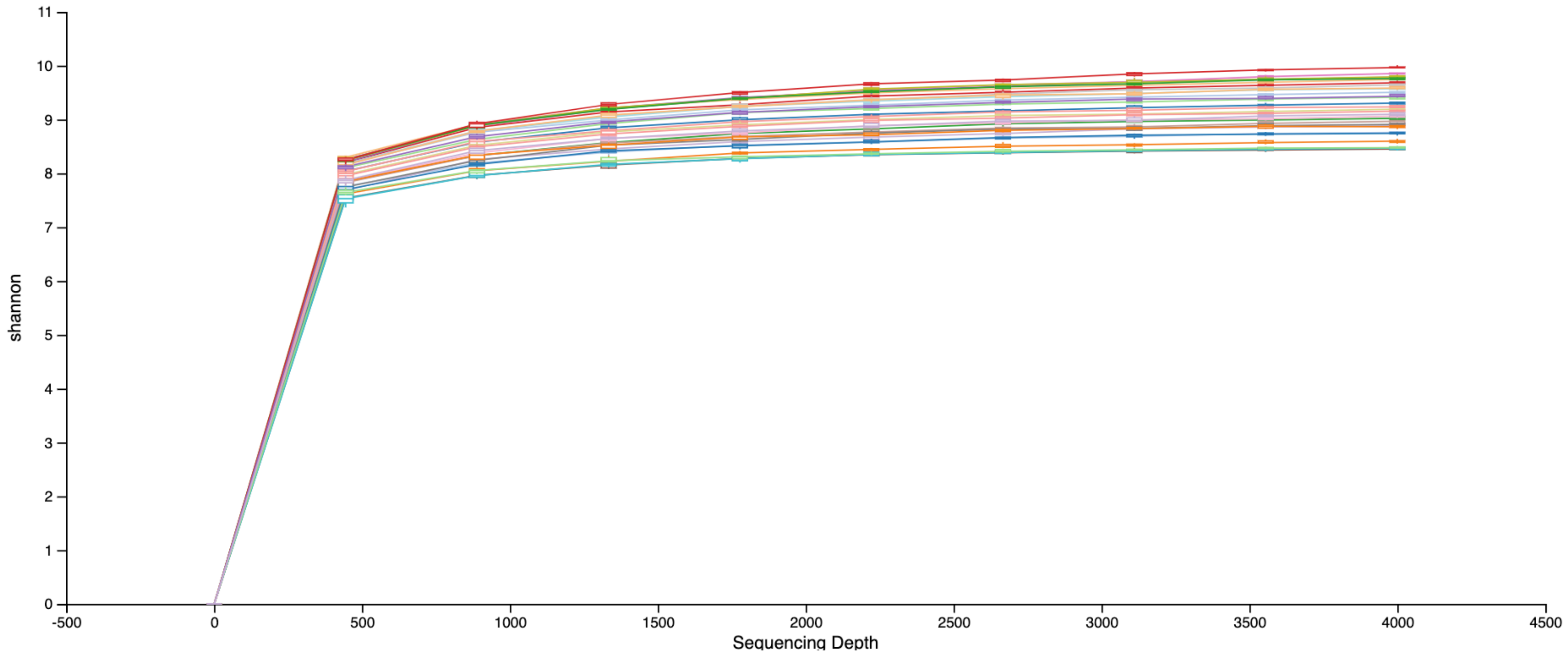

Figure S 2

Alpha Rarefaction Plot for First 16S Sequencing Run carried out in 2018. All samples have been represented with individual colors, and all curves eventually levelled out at sequencing depth shown.

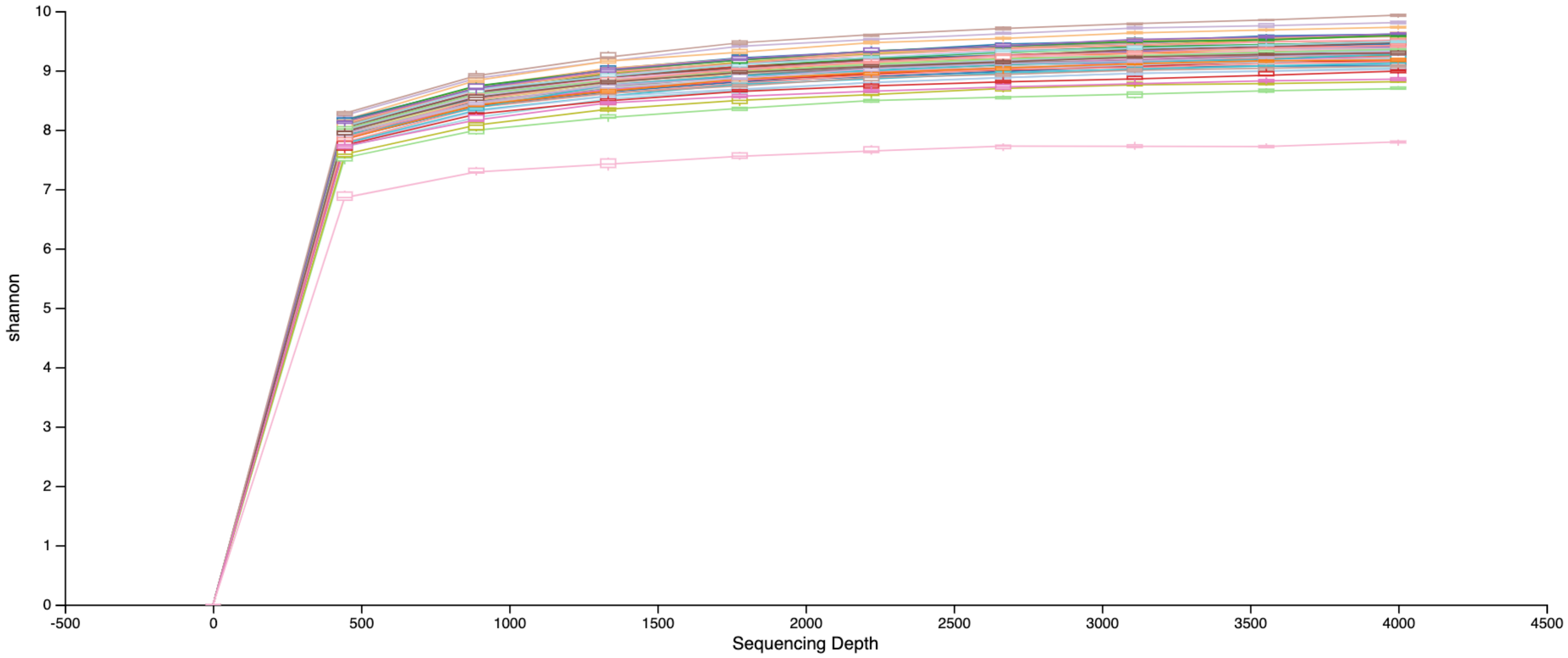

Figure S 3

Alpha Rarefaction Plot for First 16S Sequencing Run carried out in 2019. All samples have been represented with individual colors, and all curves eventually levelled out at sequencing depth shown.

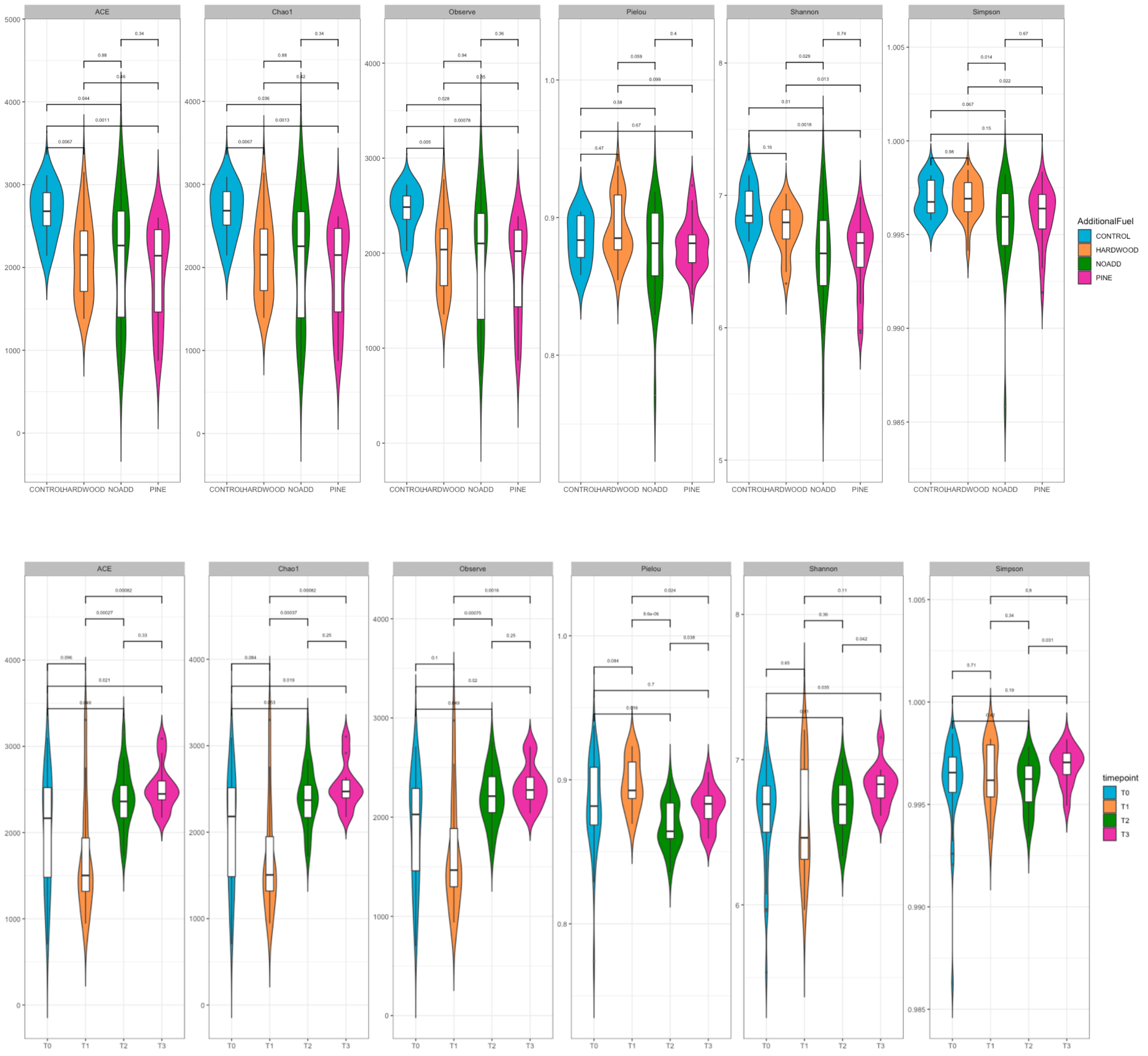

Figure S 4

Violin plots illustrating fluctuations in alpha diversity indices (ACE, Chao1, Observed, Pielou, Shannon, Simpson) across distinct slash fuel types and timepoints. Statistical significance was assessed using Wilcoxon test.

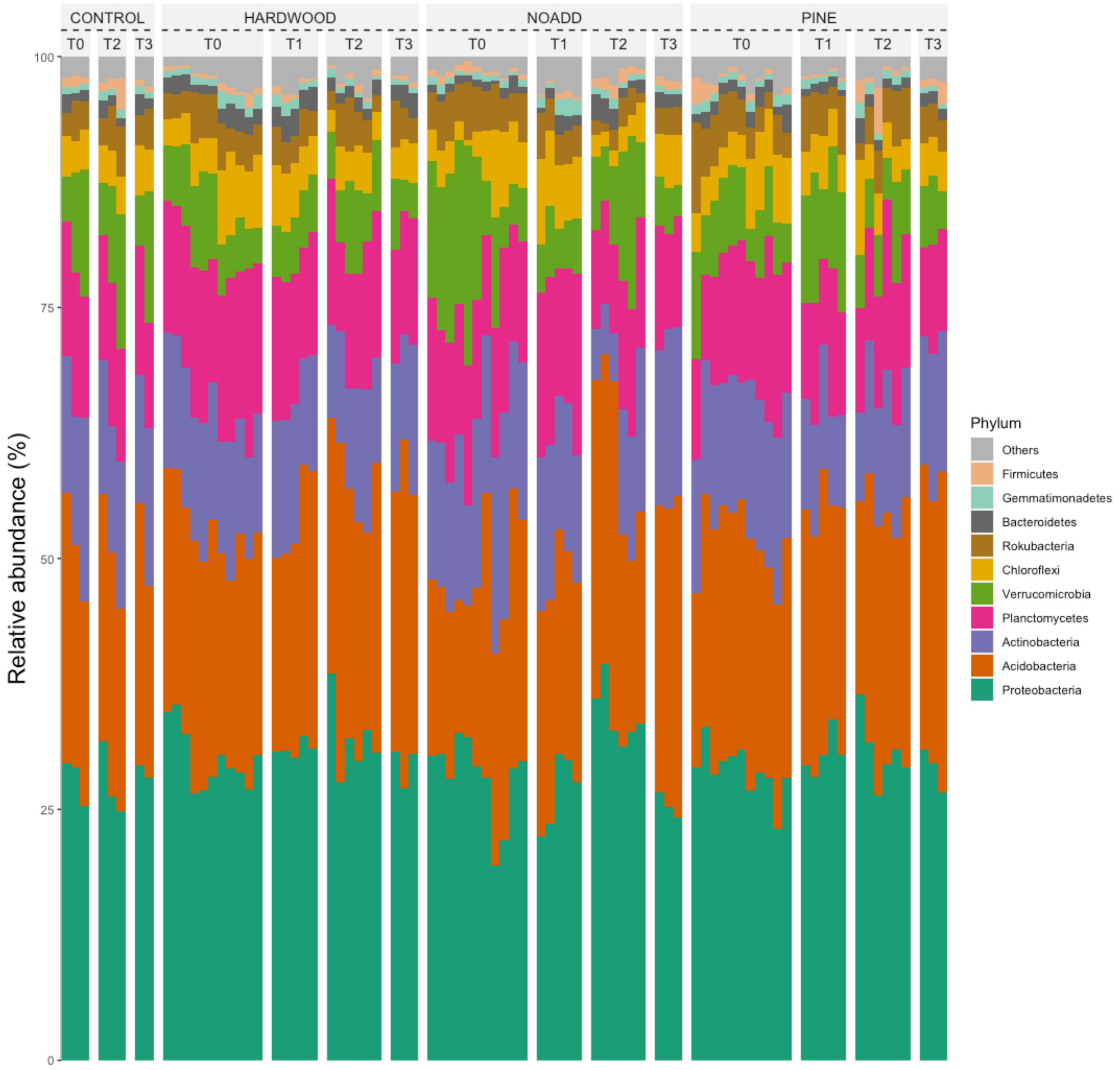

Figure S 5 Taxonomic Abundance chart. The top 10 phyla were selected to provide insights into the distribution of taxonomic groups among samples as arranged by timepoints and additional fuel treatment type.

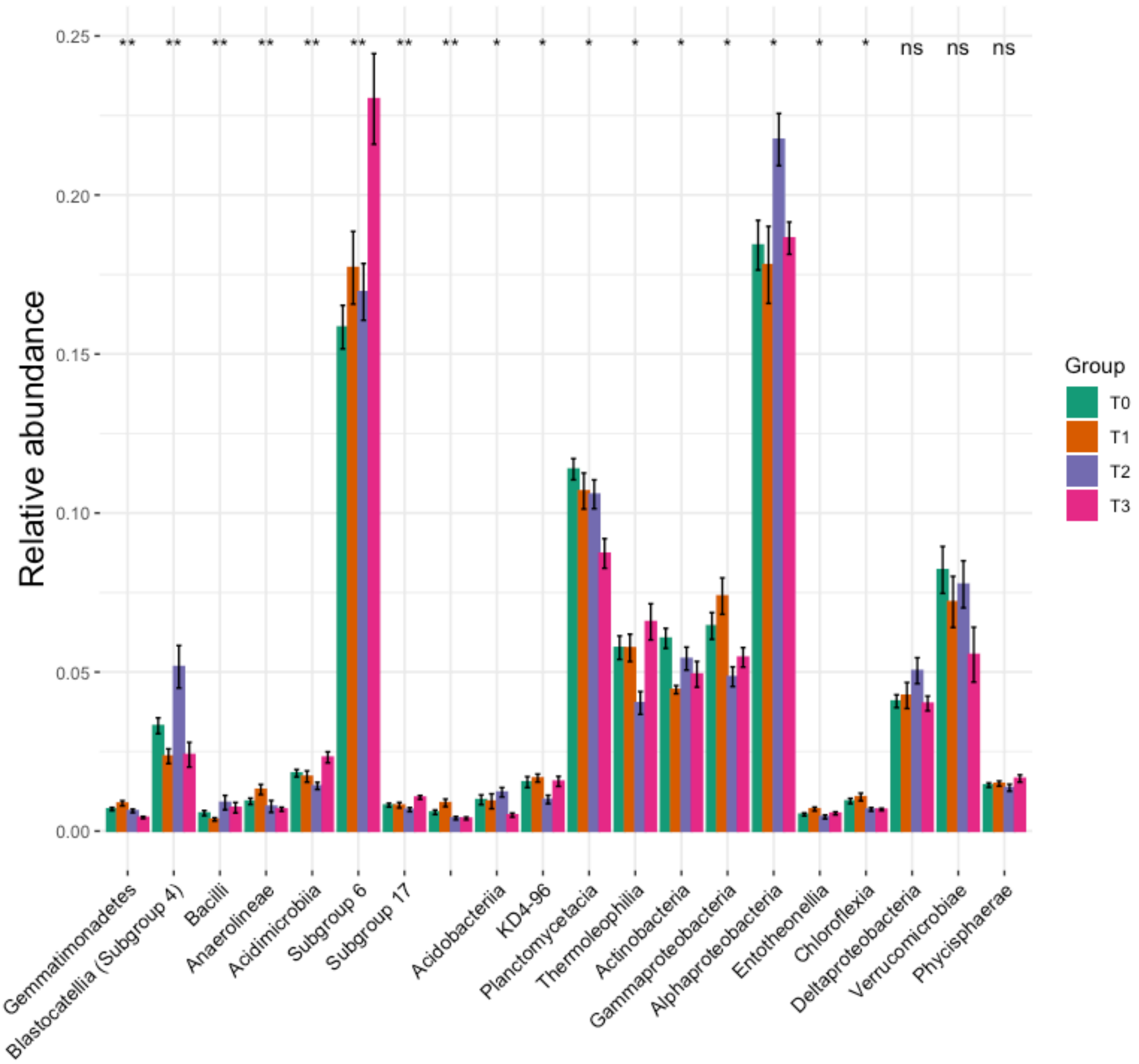

Figure S6 A

Relative Abundance plot at Class level, generated using output from the ALDEx2.kw method in the microeco package. The method performs a Kruskal-Wallis test and a glm ANOVA for the differential test metrics. Asterisks denote significant difference from the control, *P < 0.05; ** P < 0.01.

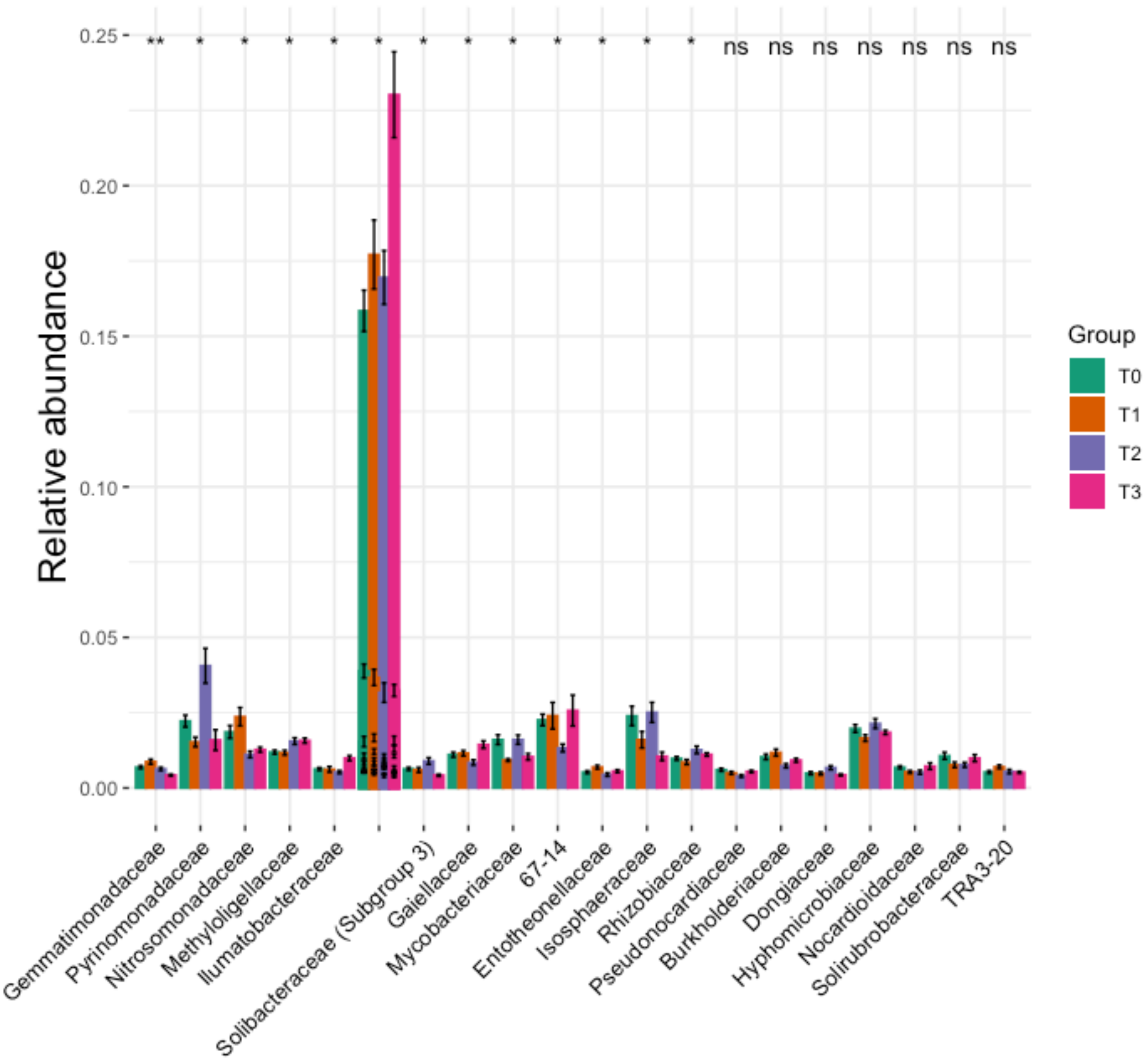

Figure S6 B

Relative Abundance plot at Family level, generated using output from the ALDEx2.kw method in the microeco package. The method performs a Kruskal-Wallis test and a glm ANOVA for the differential test metrics. Asterisks denote significant difference from the control, *P < 0.05; ** P < 0.01. Non-significant results are denoted by “ns”.

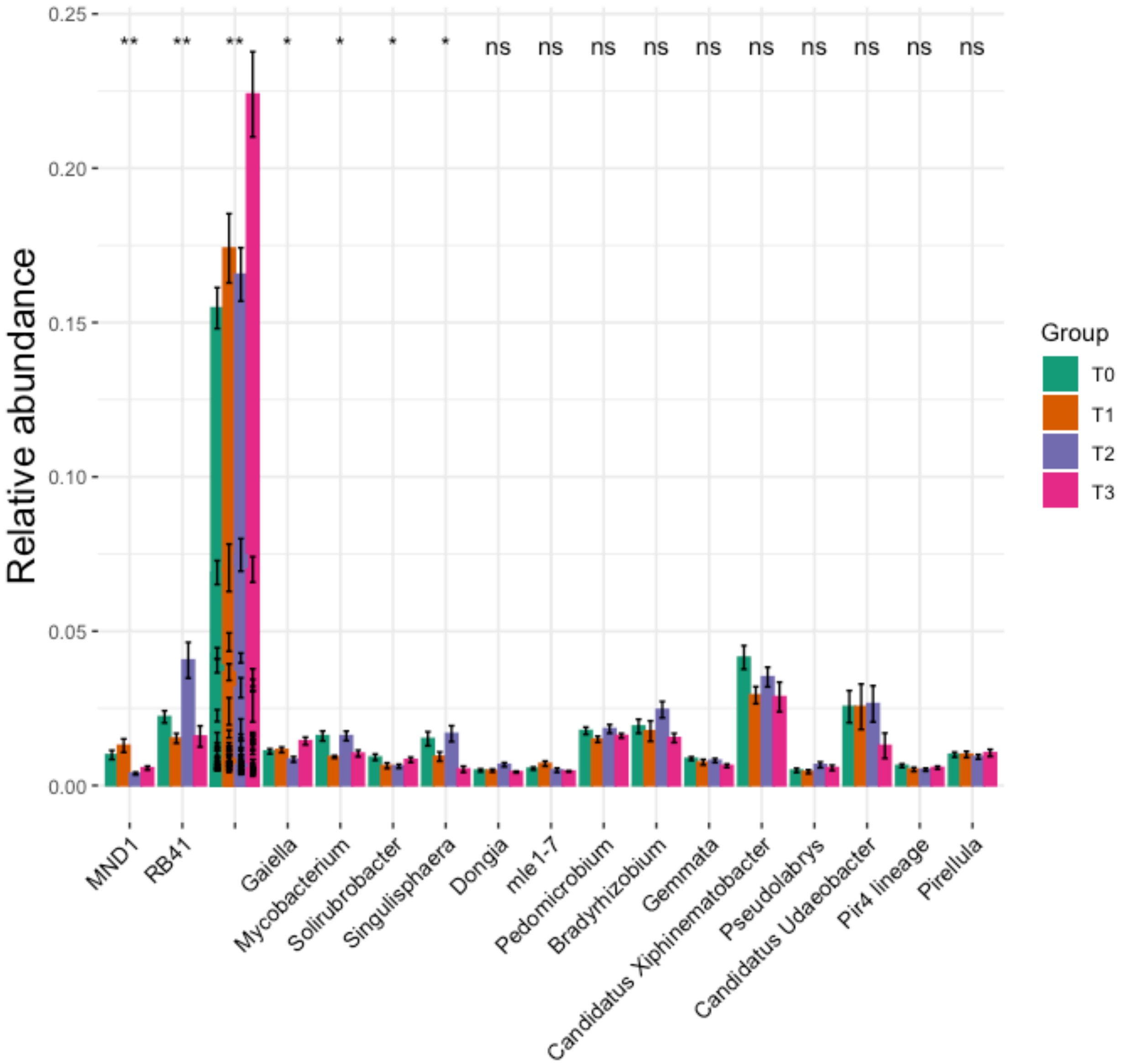

Figure S6 C

Relative Abundance plot at Genus level, generated using output from the ALDEx2.kw method in the microeco package. The method performs a Kruskal-Wallis test and a glm ANOVA for the differential test metrics. Asterisks denote significant difference from the control, *P < 0.05; ** P < 0.01. Non-significant results are denoted by “ns”.

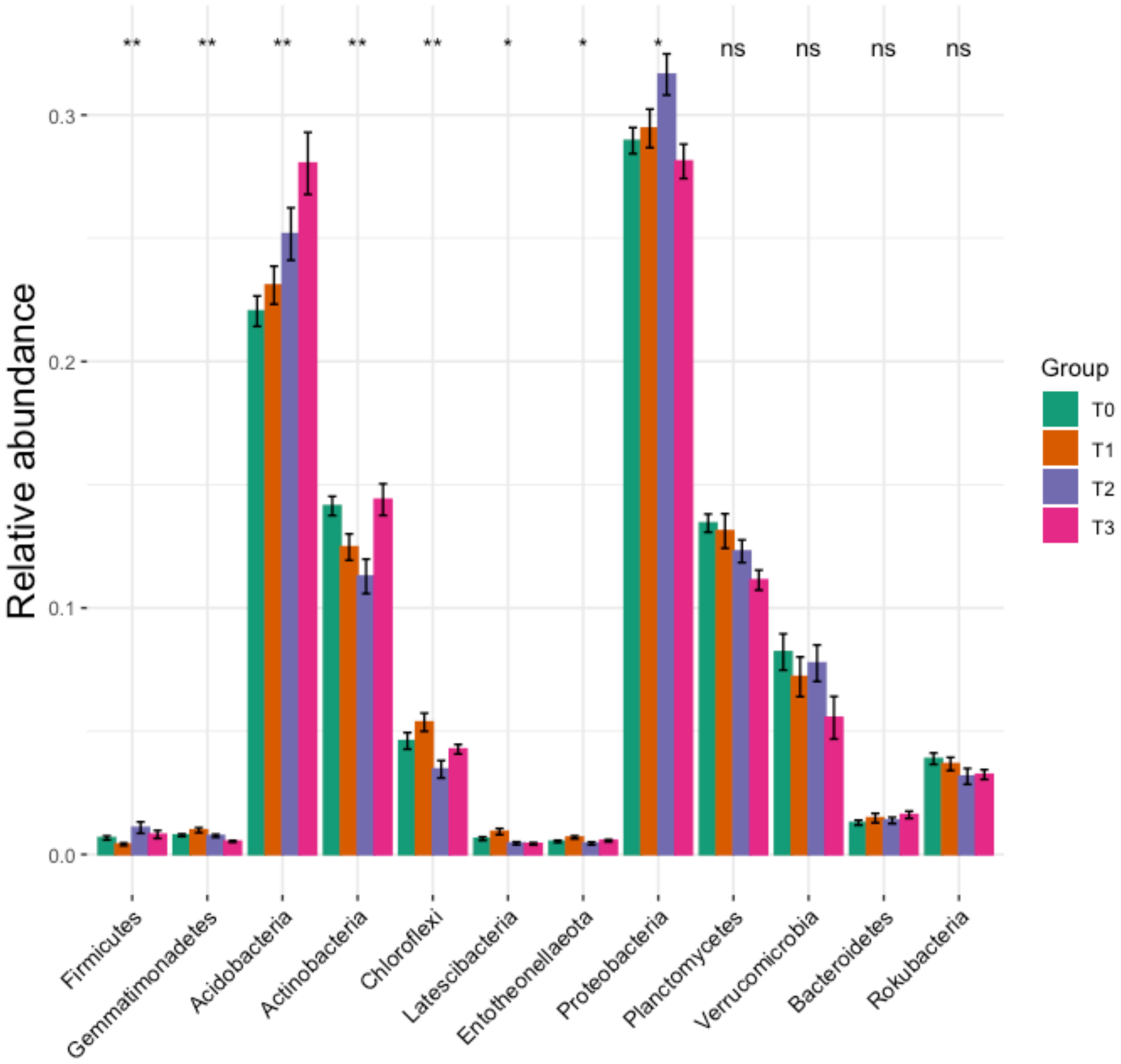

Figure S6 D

Relative Abundance plot at Phylum level, generated using output from the ALDEx2.kw method in the microeco package. The method performs a Kruskal-Wallis test and a glm ANOVA for the differential test metrics. Asterisks denote significant difference from the control, *P < 0.05; ** P < 0.01. Non-significant results are denoted by "ns".

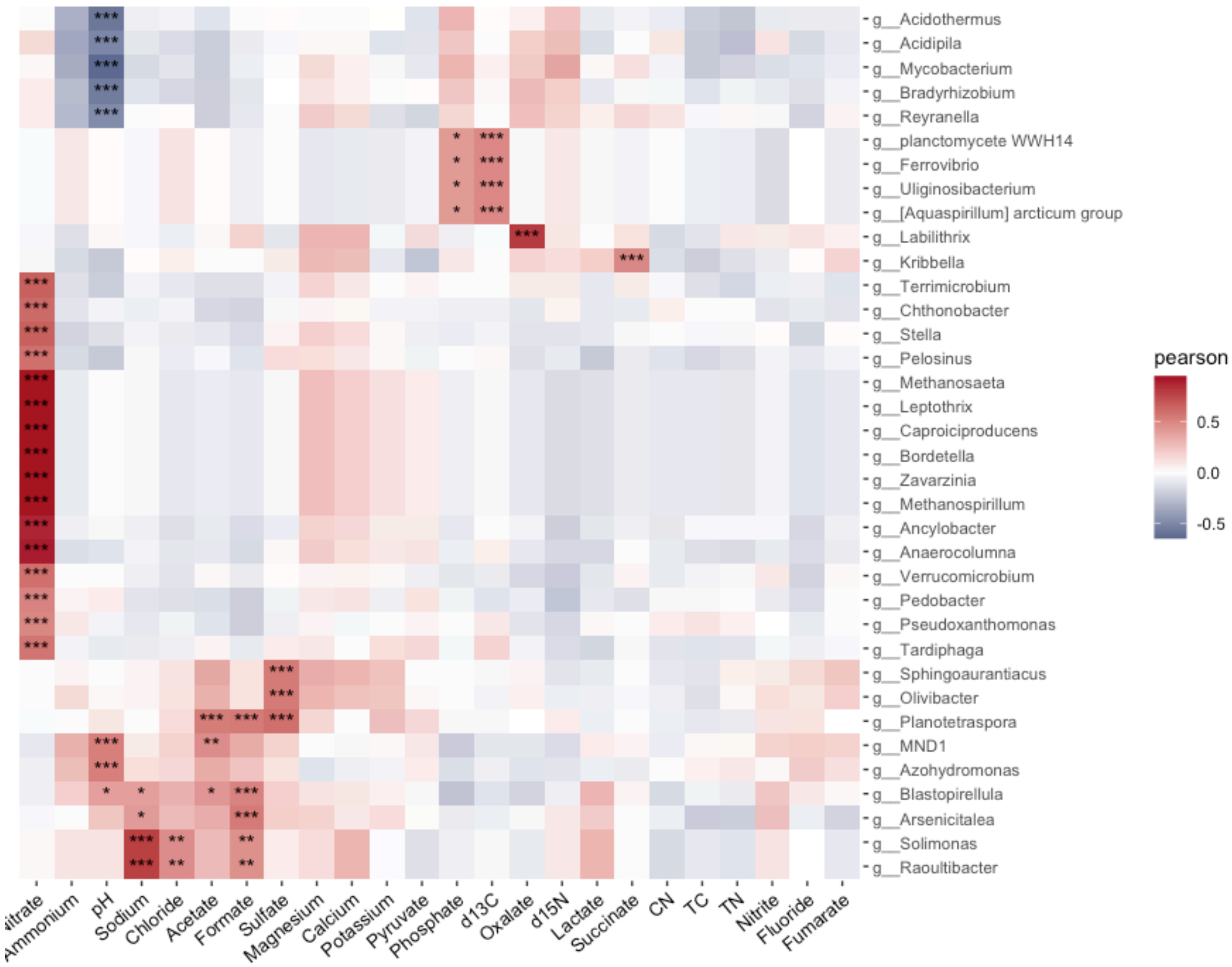

Figure S 6

Correlation heatmap depicting relationship between diverse genera and environmental parameters. Positive associations are shown in deeper red hues and negative relationships in deeper blue shades. Asterisks denote significant difference from the control, *P < 0.05; ** P < 0.01; *** P<0.001.

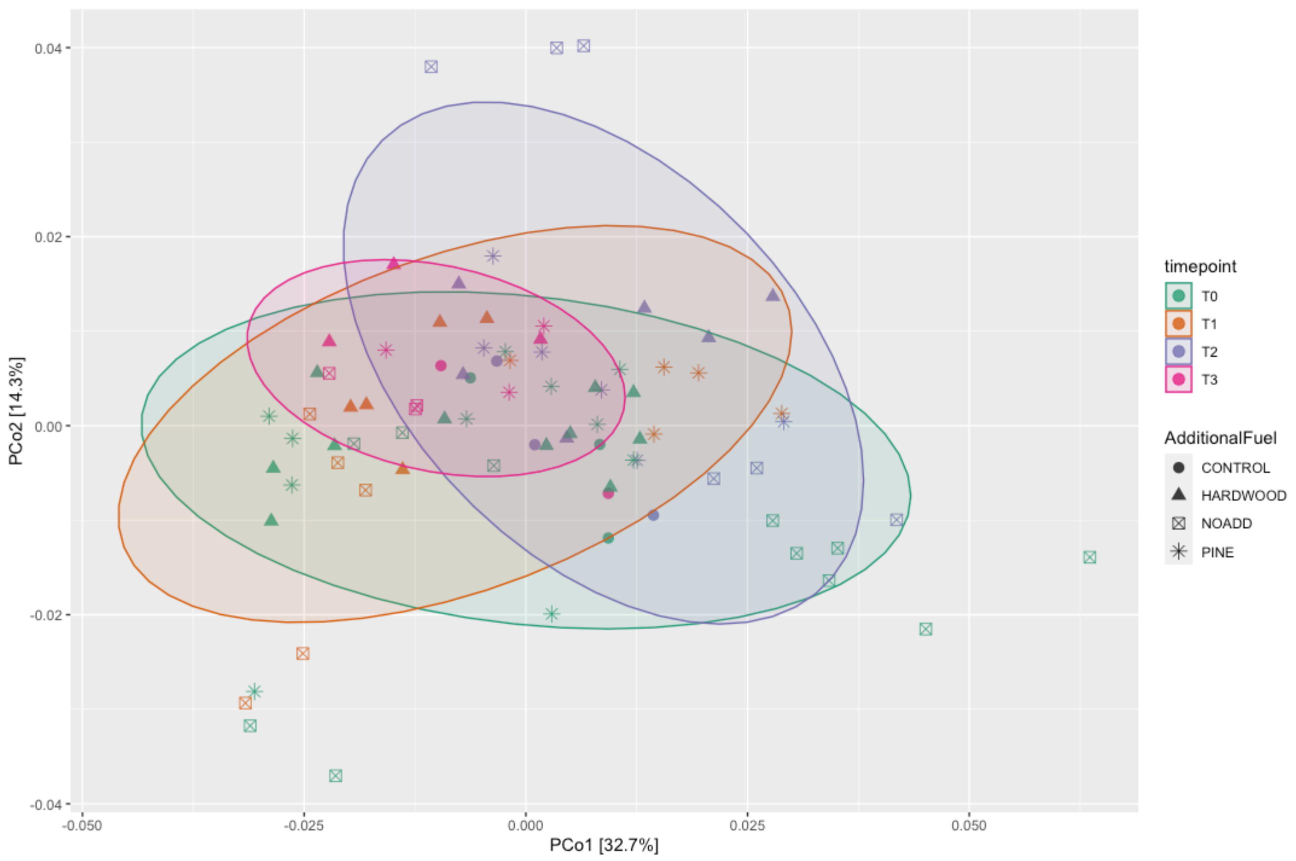

Figure S 7

Beta diversity for prokaryotic communities from soil samples. Principal coordinate analysis (PCoA) based on weighted Unifrac distances showed that time elapsed since fire explained 32.7% of variations in the soil bacterial/archaeal communities and the type of slash fuel used explained only 14.3 % of the variation. Ellipses represent standard deviation of axis scores from timepoint centroids.

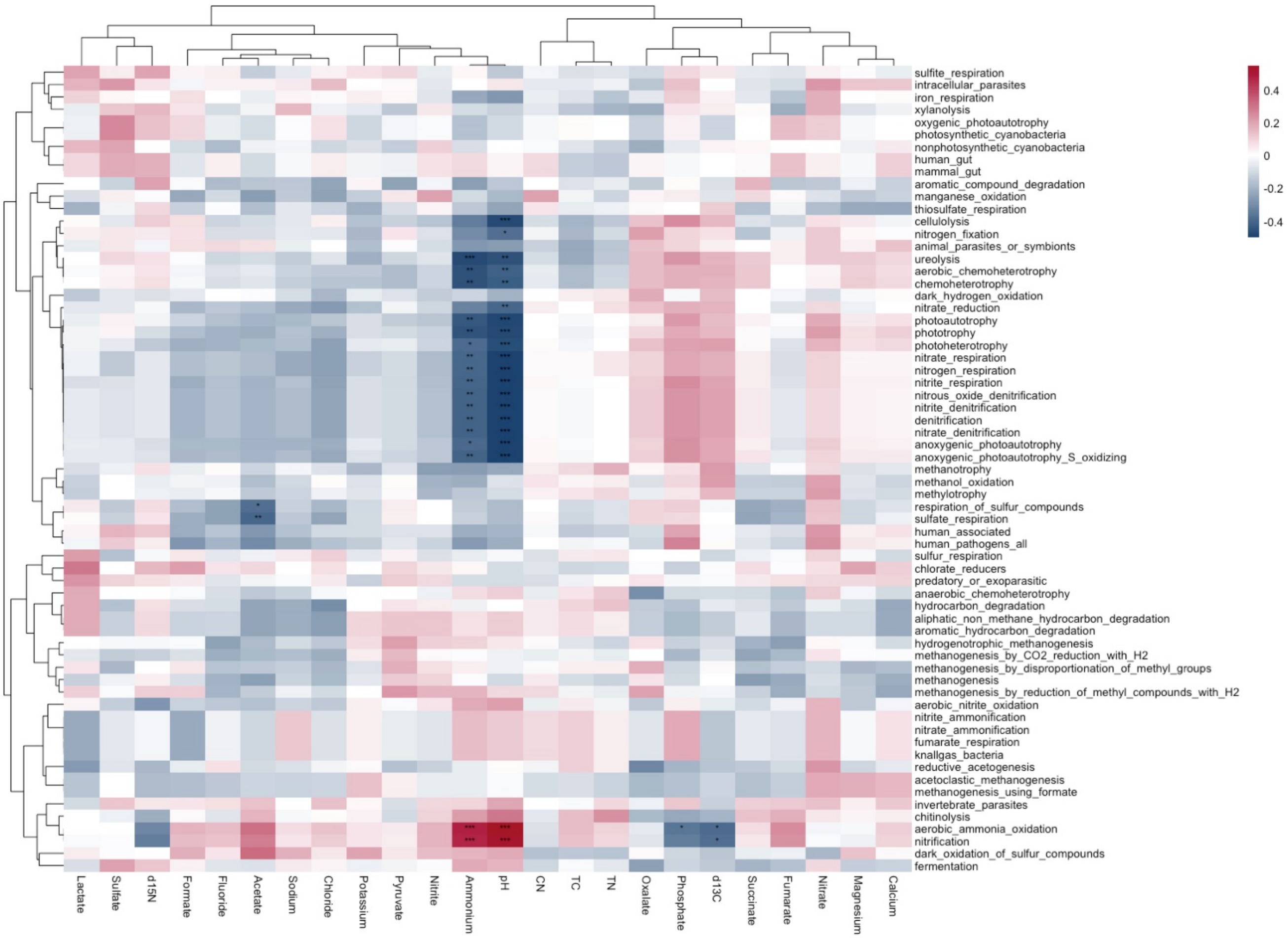

Figure S 8
FAPROTAX heat map depicting statistically significant environmental variables and their association with predicted microbial functions. The number of asterisks (*) denoting varying levels of confidence—ranging from the highest (***, $p < 0.001$, ANOVA) to the lowest (*, $p < 0.05$, ANOVA) with the color gradient indicating pearson correlation coefficient values with negative values as blue and positive values as red.

Functional Profile of Microbial Communities with 16S dataset

Significant correlations were noticed between acetate concentrations and sulfate/sulfur respiration ($p < 0.05$). As prior investigations have shown that acetate is a key substrate for sulfate respiration, it would appear. pH and NH_4^+ also exhibited a significant correlation ($p < 0.001$) with nitrogen cycling processes. This is quite understandable as pH determines the relative amounts of ammonium and ammonia ions present in the subsurface media. Fluctuations in the pH can affect the availability of these ions.

Supplementary Tables

Table T 1 showing the output of the cal_diff function in microeco package used to test the significance changes in environmental variables across groups. Significant differences are indicated by different letters ($p<0.05$, KW-Dunn) and are showed in Table T2, with the calculated P values shown here

Measure	Test_method	Group	Comparison	Z	P.unadj	P.adj	Significance
Acetate	Dunn's Kruskal-Wallis Multiple Comparisons	T0	T0 - T3	1.9984	0.0457	0.0913	ns
Acetate	Dunn's Kruskal-Wallis Multiple Comparisons	T0	T0 - T2	2.2895	0.0221	0.0662	ns
Acetate	Dunn's Kruskal-Wallis Multiple Comparisons	T1	T1 - T0	-2.3687	0.0179	0.0714	ns
Acetate	Dunn's Kruskal-Wallis Multiple Comparisons	T1	T1 - T3	3.5682	0.0004	0.0018	**
Acetate	Dunn's Kruskal-Wallis Multiple Comparisons	T1	T1 - T2	4.0128	0.0001	0.0004	***
Acetate	Dunn's Kruskal-Wallis Multiple Comparisons	T3	T3 - T2	0.1608	0.8723	0.8723	ns
Ammonium	Dunn's Kruskal-Wallis Multiple Comparisons	T0	T0 - T3	0.2442	0.8071	0.8071	ns
Ammonium	Dunn's Kruskal-Wallis Multiple Comparisons	T1	T1 - T2	0.5748	0.5655	1.0000	ns
Ammonium	Dunn's Kruskal-Wallis Multiple Comparisons	T1	T1 - T0	-1.3106	0.1900	1.0000	ns
Ammonium	Dunn's Kruskal-Wallis Multiple Comparisons	T1	T1 - T3	1.2266	0.2200	1.0000	ns
Ammonium	Dunn's Kruskal-Wallis Multiple Comparisons	T2	T2 - T0	-0.7592	0.4477	1.0000	ns
Ammonium	Dunn's Kruskal-Wallis Multiple Comparisons	T2	T2 - T3	0.7861	0.4318	1.0000	ns
Calcium	Dunn's Kruskal-Wallis Multiple Comparisons	T0	T0 - T2	0.1493	0.8813	0.8813	ns
Calcium	Dunn's Kruskal-Wallis Multiple Comparisons	T1	T1 - T3	0.7417	0.4583	1.0000	ns
Calcium	Dunn's Kruskal-Wallis Multiple Comparisons	T1	T1 - T0	-1.4112	0.1582	0.9491	ns
Calcium	Dunn's Kruskal-Wallis Multiple Comparisons	T1	T1 - T2	1.4042	0.1603	0.8013	ns
Calcium	Dunn's Kruskal-Wallis Multiple Comparisons	T3	T3 - T0	-0.4043	0.6860	1.0000	ns
Calcium	Dunn's Kruskal-Wallis Multiple Comparisons	T3	T3 - T2	-0.4844	0.6281	1.0000	ns
Chloride	Dunn's Kruskal-Wallis Multiple Comparisons	T0	T0 - T1	0.1628	0.8707	1.0000	ns
Chloride	Dunn's Kruskal-Wallis Multiple Comparisons	T0	T0 - T3	2.6802	0.0074	0.0368	*
Chloride	Dunn's Kruskal-Wallis Multiple Comparisons	T0	T0 - T2	3.4691	0.0005	0.0031	**
Chloride	Dunn's Kruskal-Wallis Multiple Comparisons	T1	T1 - T3	2.2000	0.0278	0.0834	ns
Chloride	Dunn's Kruskal-Wallis Multiple Comparisons	T1	T1 - T2	2.6697	0.0076	0.0304	*

Chloride	Dunn's Kruskal-Wallis Multiple Comparisons	T3	T3 - T2	-0.0785	0.9375	0.9375	ns
CN	Dunn's Kruskal-Wallis Multiple Comparisons	T0	T0 - T3	0.0844	0.9327	0.9327	ns
CN	Dunn's Kruskal-Wallis Multiple Comparisons	T0	T0 - T1	1.3812	0.1672	0.8361	ns
CN	Dunn's Kruskal-Wallis Multiple Comparisons	T2	T2 - T0	-1.1763	0.2395	0.9579	ns
CN	Dunn's Kruskal-Wallis Multiple Comparisons	T2	T2 - T3	0.9460	0.3441	0.6883	ns
CN	Dunn's Kruskal-Wallis Multiple Comparisons	T2	T2 - T1	-2.2111	0.0270	0.1622	ns
CN	Dunn's Kruskal-Wallis Multiple Comparisons	T3	T3 - T1	-0.9960	0.3192	0.9577	ns
d13C	Dunn's Kruskal-Wallis Multiple Comparisons	T0	T0 - T1	1.7168	0.0860	0.4301	ns
d13C	Dunn's Kruskal-Wallis Multiple Comparisons	T0	T0 - T2	1.1460	0.2518	0.7554	ns
d13C	Dunn's Kruskal-Wallis Multiple Comparisons	T0	T0 - T3	2.4298	0.0151	0.0906	ns
d13C	Dunn's Kruskal-Wallis Multiple Comparisons	T1	T1 - T2	-0.6298	0.5288	0.5288	ns
d13C	Dunn's Kruskal-Wallis Multiple Comparisons	T1	T1 - T3	0.7797	0.4356	0.8712	ns
d13C	Dunn's Kruskal-Wallis Multiple Comparisons	T2	T2 - T3	1.4036	0.1604	0.6417	ns
d15N	Dunn's Kruskal-Wallis Multiple Comparisons	T0	T0 - T2	1.0153	0.3100	0.9299	ns
d15N	Dunn's Kruskal-Wallis Multiple Comparisons	T0	T0 - T1	2.3268	0.0200	0.1198	ns
d15N	Dunn's Kruskal-Wallis Multiple Comparisons	T0	T0 - T3	1.8392	0.0659	0.3294	ns
d15N	Dunn's Kruskal-Wallis Multiple Comparisons	T1	T1 - T3	-0.2052	0.8374	0.8374	ns
d15N	Dunn's Kruskal-Wallis Multiple Comparisons	T2	T2 - T1	-1.2906	0.1969	0.7874	ns
d15N	Dunn's Kruskal-Wallis Multiple Comparisons	T2	T2 - T3	0.9534	0.3404	0.6808	ns
Fluoride	Dunn's Kruskal-Wallis Multiple Comparisons	T0	T0 - T3	0.2456	0.8060	1.0000	ns
Fluoride	Dunn's Kruskal-Wallis Multiple Comparisons	T0	T0 - T1	-0.1824	0.8553	0.8553	ns
Fluoride	Dunn's Kruskal-Wallis Multiple Comparisons	T0	T0 - T2	2.4243	0.0153	0.0920	ns
Fluoride	Dunn's Kruskal-Wallis Multiple Comparisons	T1	T1 - T2	2.1348	0.0328	0.1639	ns
Fluoride	Dunn's Kruskal-Wallis Multiple Comparisons	T3	T3 - T1	0.3543	0.7231	1.0000	ns
Fluoride	Dunn's Kruskal-Wallis Multiple Comparisons	T3	T3 - T2	-1.5612	0.1185	0.4739	ns
Formate	Dunn's Kruskal-Wallis Multiple Comparisons	T0	T0 - T2	2.5438	0.0110	0.0329	*
Formate	Dunn's Kruskal-Wallis Multiple Comparisons	T0	T0 - T3	3.3312	0.0009	0.0043	**
Formate	Dunn's Kruskal-Wallis Multiple Comparisons	T1	T1 - T0	-1.1559	0.2477	0.2477	ns
Formate	Dunn's Kruskal-Wallis Multiple Comparisons	T1	T1 - T2	3.1170	0.0018	0.0073	**
Formate	Dunn's Kruskal-Wallis Multiple Comparisons	T1	T1 - T3	3.7859	0.0002	0.0009	***

Formate	Dunn's Kruskal-Wallis Multiple Comparisons	T2	T2 - T3	1.2067	0.2275	0.4551	ns
Fumarate	Dunn's Kruskal-Wallis Multiple Comparisons	T0	T0 - T1	1.3348	0.1819	0.7277	ns
Fumarate	Dunn's Kruskal-Wallis Multiple Comparisons	T0	T0 - T2	2.5386	0.0111	0.0668	ns
Fumarate	Dunn's Kruskal-Wallis Multiple Comparisons	T0	T0 - T3	2.3046	0.0212	0.1059	ns
Fumarate	Dunn's Kruskal-Wallis Multiple Comparisons	T1	T1 - T2	0.8485	0.3962	0.7923	ns
Fumarate	Dunn's Kruskal-Wallis Multiple Comparisons	T1	T1 - T3	0.9667	0.3337	1.0000	ns
Fumarate	Dunn's Kruskal-Wallis Multiple Comparisons	T2	T2 - T3	0.2604	0.7946	0.7946	ns
Lactate	Dunn's Kruskal-Wallis Multiple Comparisons	T0	T0 - T3	0.3977	0.6908	0.6908	ns
Lactate	Dunn's Kruskal-Wallis Multiple Comparisons	T1	T1 - T0	-0.6566	0.5115	1.0000	ns
Lactate	Dunn's Kruskal-Wallis Multiple Comparisons	T1	T1 - T3	0.8535	0.3934	1.0000	ns
Lactate	Dunn's Kruskal-Wallis Multiple Comparisons	T2	T2 - T1	-0.7874	0.4310	1.0000	ns
Lactate	Dunn's Kruskal-Wallis Multiple Comparisons	T2	T2 - T0	-1.7043	0.0883	0.5300	ns
Lactate	Dunn's Kruskal-Wallis Multiple Comparisons	T2	T2 - T3	1.6255	0.1041	0.5203	ns
Magnesium	Dunn's Kruskal-Wallis Multiple Comparisons	T0	T0 - T2	1.9354	0.0529	0.2647	ns
Magnesium	Dunn's Kruskal-Wallis Multiple Comparisons	T0	T0 - T3	2.0464	0.0407	0.2443	ns
Magnesium	Dunn's Kruskal-Wallis Multiple Comparisons	T1	T1 - T0	-0.1388	0.8896	0.8896	ns
Magnesium	Dunn's Kruskal-Wallis Multiple Comparisons	T1	T1 - T2	1.6982	0.0895	0.2684	ns
Magnesium	Dunn's Kruskal-Wallis Multiple Comparisons	T1	T1 - T3	1.8834	0.0596	0.2386	ns
Magnesium	Dunn's Kruskal-Wallis Multiple Comparisons	T2	T2 - T3	0.4663	0.6410	1.0000	ns
Nitrate	Dunn's Kruskal-Wallis Multiple Comparisons	T0	T0 - T3	1.3025	0.1927	0.3855	ns
Nitrate	Dunn's Kruskal-Wallis Multiple Comparisons	T1	T1 - T0	-1.5645	0.1177	0.3531	ns
Nitrate	Dunn's Kruskal-Wallis Multiple Comparisons	T1	T1 - T3	2.3416	0.0192	0.0960	ns
Nitrate	Dunn's Kruskal-Wallis Multiple Comparisons	T2	T2 - T1	-0.4005	0.6888	0.6888	ns
Nitrate	Dunn's Kruskal-Wallis Multiple Comparisons	T2	T2 - T0	-2.2441	0.0248	0.0993	ns
Nitrate	Dunn's Kruskal-Wallis Multiple Comparisons	T2	T2 - T3	2.8612	0.0042	0.0253	*
Nitrite	Dunn's Kruskal-Wallis Multiple Comparisons	T0	T0 - T1	-1.1204	0.2625	1.0000	ns
Nitrite	Dunn's Kruskal-Wallis Multiple Comparisons	T2	T2 - T0	-1.7494	0.0802	0.4011	ns
Nitrite	Dunn's Kruskal-Wallis Multiple Comparisons	T2	T2 - T1	-0.4024	0.6874	1.0000	ns
Nitrite	Dunn's Kruskal-Wallis Multiple Comparisons	T3	T3 - T2	-0.3700	0.7114	0.7114	ns
Nitrite	Dunn's Kruskal-Wallis Multiple Comparisons	T3	T3 - T0	-1.7940	0.0728	0.4369	ns
Nitrite	Dunn's Kruskal-Wallis Multiple Comparisons	T3	T3 - T1	-0.6896	0.4905	1.0000	ns

Oxalate	Dunn's Kruskal-Wallis Multiple Comparisons	T0	T0 - T2	1.5879	0.1123	0.3370	ns
Oxalate	Dunn's Kruskal-Wallis Multiple Comparisons	T0	T0 - T3	2.6533	0.0080	0.0399	*
Oxalate	Dunn's Kruskal-Wallis Multiple Comparisons	T1	T1 - T0	-0.8811	0.3783	0.3783	ns
Oxalate	Dunn's Kruskal-Wallis Multiple Comparisons	T1	T1 - T2	2.0907	0.0366	0.1462	ns
Oxalate	Dunn's Kruskal-Wallis Multiple Comparisons	T1	T1 - T3	2.9848	0.0028	0.0170	*
Oxalate	Dunn's Kruskal-Wallis Multiple Comparisons	T2	T2 - T3	1.2845	0.1990	0.3980	ns
pH	Dunn's Kruskal-Wallis Multiple Comparisons	T1	T1 - T2	0.9174	0.3590	0.7179	ns
pH	Dunn's Kruskal-Wallis Multiple Comparisons	T1	T1 - T3	1.5580	0.1192	0.4769	ns
pH	Dunn's Kruskal-Wallis Multiple Comparisons	T1	T1 - T0	-3.5920	0.0003	0.0020	**
pH	Dunn's Kruskal-Wallis Multiple Comparisons	T2	T2 - T3	0.8285	0.4074	0.4074	ns
pH	Dunn's Kruskal-Wallis Multiple Comparisons	T2	T2 - T0	-2.8908	0.0038	0.0192	*
pH	Dunn's Kruskal-Wallis Multiple Comparisons	T3	T3 - T0	-1.4090	0.1588	0.4765	ns
Phosphate	Dunn's Kruskal-Wallis Multiple Comparisons	T0	T0 - T3	1.3846	0.1662	0.9971	ns
Phosphate	Dunn's Kruskal-Wallis Multiple Comparisons	T0	T0 - T1	1.2709	0.2038	1.0000	ns
Phosphate	Dunn's Kruskal-Wallis Multiple Comparisons	T0	T0 - T2	1.1751	0.2400	0.9598	ns
Phosphate	Dunn's Kruskal-Wallis Multiple Comparisons	T1	T1 - T2	-0.2009	0.8408	0.8408	ns
Phosphate	Dunn's Kruskal-Wallis Multiple Comparisons	T3	T3 - T1	0.2177	0.8276	1.0000	ns
Phosphate	Dunn's Kruskal-Wallis Multiple Comparisons	T3	T3 - T2	0.4147	0.6784	1.0000	ns
Potassium	Dunn's Kruskal-Wallis Multiple Comparisons	T1	T1 - T3	-0.6995	0.4842	0.4842	ns
Potassium	Dunn's Kruskal-Wallis Multiple Comparisons	T1	T1 - T2	1.0036	0.3156	0.6311	ns
Potassium	Dunn's Kruskal-Wallis Multiple Comparisons	T1	T1 - T0	-2.3628	0.0181	0.0907	ns
Potassium	Dunn's Kruskal-Wallis Multiple Comparisons	T2	T2 - T0	-1.4089	0.1589	0.4766	ns
Potassium	Dunn's Kruskal-Wallis Multiple Comparisons	T3	T3 - T2	-1.6576	0.0974	0.3896	ns
Potassium	Dunn's Kruskal-Wallis Multiple Comparisons	T3	T3 - T0	-2.9138	0.0036	0.0214	*
Pyruvate	Dunn's Kruskal-Wallis Multiple Comparisons	T0	T0 - T3	0.3965	0.6917	1.0000	ns
Pyruvate	Dunn's Kruskal-Wallis Multiple Comparisons	T1	T1 - T0	-0.2796	0.7798	0.7798	ns
Pyruvate	Dunn's Kruskal-Wallis Multiple Comparisons	T1	T1 - T3	0.5605	0.5751	1.0000	ns
Pyruvate	Dunn's Kruskal-Wallis Multiple Comparisons	T2	T2 - T1	-1.3644	0.1724	0.6897	ns
Pyruvate	Dunn's Kruskal-Wallis Multiple Comparisons	T2	T2 - T0	-1.9927	0.0463	0.2777	ns
Pyruvate	Dunn's Kruskal-Wallis Multiple Comparisons	T2	T2 - T3	1.8372	0.0662	0.3309	ns

Sodium	Dunn's Kruskal-Wallis Multiple Comparisons	T0	T0 - T3	0.5100	0.6101	0.6101	ns
Sodium	Dunn's Kruskal-Wallis Multiple Comparisons	T0	T0 - T2	3.1413	0.0017	0.0084	**
Sodium	Dunn's Kruskal-Wallis Multiple Comparisons	T1	T1 - T0	-1.1512	0.2496	0.4993	ns
Sodium	Dunn's Kruskal-Wallis Multiple Comparisons	T1	T1 - T3	1.3339	0.1822	0.5467	ns
Sodium	Dunn's Kruskal-Wallis Multiple Comparisons	T1	T1 - T2	3.5980	0.0003	0.0019	**
Sodium	Dunn's Kruskal-Wallis Multiple Comparisons	T3	T3 - T2	-1.8454	0.0650	0.2599	ns
Succinate	Dunn's Kruskal-Wallis Multiple Comparisons	T0	T0 - T2	-0.2781	0.7809	0.7809	ns
Succinate	Dunn's Kruskal-Wallis Multiple Comparisons	T0	T0 - T3	0.7206	0.4711	1.0000	ns
Succinate	Dunn's Kruskal-Wallis Multiple Comparisons	T1	T1 - T0	-0.6395	0.5225	1.0000	ns
Succinate	Dunn's Kruskal-Wallis Multiple Comparisons	T1	T1 - T2	0.3554	0.7223	1.0000	ns
Succinate	Dunn's Kruskal-Wallis Multiple Comparisons	T1	T1 - T3	1.1205	0.2625	1.0000	ns
Succinate	Dunn's Kruskal-Wallis Multiple Comparisons	T2	T2 - T3	0.8722	0.3831	1.0000	ns
Sulfate	Dunn's Kruskal-Wallis Multiple Comparisons	T0	T0 - T3	1.6061	0.1083	0.5413	ns
Sulfate	Dunn's Kruskal-Wallis Multiple Comparisons	T1	T1 - T2	1.4238	0.1545	0.3090	ns
Sulfate	Dunn's Kruskal-Wallis Multiple Comparisons	T1	T1 - T0	-1.5027	0.1329	0.5317	ns
Sulfate	Dunn's Kruskal-Wallis Multiple Comparisons	T1	T1 - T3	2.5572	0.0106	0.0633	ns
Sulfate	Dunn's Kruskal-Wallis Multiple Comparisons	T2	T2 - T0	0.0711	0.9433	0.9433	ns
Sulfate	Dunn's Kruskal-Wallis Multiple Comparisons	T2	T2 - T3	1.4342	0.1515	0.4546	ns
TC	Dunn's Kruskal-Wallis Multiple Comparisons	T0	T0 - T1	0.7489	0.4539	0.9079	ns
TC	Dunn's Kruskal-Wallis Multiple Comparisons	T2	T2 - T3	0.1657	0.8684	0.8684	ns
TC	Dunn's Kruskal-Wallis Multiple Comparisons	T2	T2 - T0	-1.5833	0.1134	0.5668	ns
TC	Dunn's Kruskal-Wallis Multiple Comparisons	T2	T2 - T1	-1.9668	0.0492	0.2953	ns
TC	Dunn's Kruskal-Wallis Multiple Comparisons	T3	T3 - T0	-1.0829	0.2788	0.8365	ns
TC	Dunn's Kruskal-Wallis Multiple Comparisons	T3	T3 - T1	-1.5196	0.1286	0.5144	ns
TN	Dunn's Kruskal-Wallis Multiple Comparisons	T0	T0 - T1	0.7126	0.4761	0.9521	ns
TN	Dunn's Kruskal-Wallis Multiple Comparisons	T2	T2 - T0	-0.7645	0.4446	1.0000	ns
TN	Dunn's Kruskal-Wallis Multiple Comparisons	T2	T2 - T1	-1.2688	0.2045	1.0000	ns
TN	Dunn's Kruskal-Wallis Multiple Comparisons	T3	T3 - T2	-0.3448	0.7303	0.7303	ns
TN	Dunn's Kruskal-Wallis Multiple Comparisons	T3	T3 - T0	-0.9818	0.3262	1.0000	ns
TN	Dunn's Kruskal-Wallis Multiple Comparisons	T3	T3 - T1	-1.4038	0.1604	0.9622	ns

Table T 2 shows the Letter Display output for the multiple comparison significance testing on environmental variables. Different letters represent significant differences.

Measure	Test_method	Group	Letter	MonoLetter
Acetate	Dunn's Kruskal-Wallis Multiple Comparisons	T0	ab	ab
Acetate	Dunn's Kruskal-Wallis Multiple Comparisons	T1	a	a
Acetate	Dunn's Kruskal-Wallis Multiple Comparisons	T2	b	b
Acetate	Dunn's Kruskal-Wallis Multiple Comparisons	T3	b	b
Ammonium	Dunn's Kruskal-Wallis Multiple Comparisons	T0	a	a
Ammonium	Dunn's Kruskal-Wallis Multiple Comparisons	T1	a	a
Ammonium	Dunn's Kruskal-Wallis Multiple Comparisons	T2	a	a
Ammonium	Dunn's Kruskal-Wallis Multiple Comparisons	T3	a	a
Calcium	Dunn's Kruskal-Wallis Multiple Comparisons	T0	a	a
Calcium	Dunn's Kruskal-Wallis Multiple Comparisons	T1	a	a
Calcium	Dunn's Kruskal-Wallis Multiple Comparisons	T2	a	a
Calcium	Dunn's Kruskal-Wallis Multiple Comparisons	T3	a	a
Chloride	Dunn's Kruskal-Wallis Multiple Comparisons	T0	a	a
Chloride	Dunn's Kruskal-Wallis Multiple Comparisons	T1	ab	ab
Chloride	Dunn's Kruskal-Wallis Multiple Comparisons	T2	c	c
Chloride	Dunn's Kruskal-Wallis Multiple Comparisons	T3	bc	bc
CN	Dunn's Kruskal-Wallis Multiple Comparisons	T0	a	a
CN	Dunn's Kruskal-Wallis Multiple Comparisons	T1	a	a
CN	Dunn's Kruskal-Wallis Multiple Comparisons	T2	a	a
CN	Dunn's Kruskal-Wallis Multiple Comparisons	T3	a	a
d13C	Dunn's Kruskal-Wallis Multiple Comparisons	T0	a	a
d13C	Dunn's Kruskal-Wallis Multiple Comparisons	T1	a	a
d13C	Dunn's Kruskal-Wallis Multiple Comparisons	T2	a	a

d13C	Dunn's Kruskal-Wallis Multiple Comparisons	T3	a	a
d15N	Dunn's Kruskal-Wallis Multiple Comparisons	T0	a	a
d15N	Dunn's Kruskal-Wallis Multiple Comparisons	T1	a	a
d15N	Dunn's Kruskal-Wallis Multiple Comparisons	T2	a	a
d15N	Dunn's Kruskal-Wallis Multiple Comparisons	T3	a	a
Fluoride	Dunn's Kruskal-Wallis Multiple Comparisons	T0	a	a
Fluoride	Dunn's Kruskal-Wallis Multiple Comparisons	T1	a	a
Fluoride	Dunn's Kruskal-Wallis Multiple Comparisons	T2	a	a
Fluoride	Dunn's Kruskal-Wallis Multiple Comparisons	T3	a	a
Formate	Dunn's Kruskal-Wallis Multiple Comparisons	T0	a	a
Formate	Dunn's Kruskal-Wallis Multiple Comparisons	T1	a	a
Formate	Dunn's Kruskal-Wallis Multiple Comparisons	T2	b	b
Formate	Dunn's Kruskal-Wallis Multiple Comparisons	T3	b	b
Fumarate	Dunn's Kruskal-Wallis Multiple Comparisons	T0	a	a
Fumarate	Dunn's Kruskal-Wallis Multiple Comparisons	T1	a	a
Fumarate	Dunn's Kruskal-Wallis Multiple Comparisons	T2	a	a
Fumarate	Dunn's Kruskal-Wallis Multiple Comparisons	T3	a	a
Lactate	Dunn's Kruskal-Wallis Multiple Comparisons	T0	a	a
Lactate	Dunn's Kruskal-Wallis Multiple Comparisons	T1	a	a
Lactate	Dunn's Kruskal-Wallis Multiple Comparisons	T2	a	a
Lactate	Dunn's Kruskal-Wallis Multiple Comparisons	T3	a	a
Magnesium	Dunn's Kruskal-Wallis Multiple Comparisons	T0	a	a
Magnesium	Dunn's Kruskal-Wallis Multiple Comparisons	T1	a	a
Magnesium	Dunn's Kruskal-Wallis Multiple Comparisons	T2	a	a
Magnesium	Dunn's Kruskal-Wallis Multiple Comparisons	T3	a	a
Nitrate	Dunn's Kruskal-Wallis Multiple Comparisons	T0	ab	ab
Nitrate	Dunn's Kruskal-Wallis Multiple Comparisons	T1	ab	ab
Nitrate	Dunn's Kruskal-Wallis Multiple Comparisons	T2	a	a

Nitrate	Dunn's Kruskal-Wallis Multiple Comparisons	T3	b	b
Nitrite	Dunn's Kruskal-Wallis Multiple Comparisons	T0	a	a
Nitrite	Dunn's Kruskal-Wallis Multiple Comparisons	T1	a	a
Nitrite	Dunn's Kruskal-Wallis Multiple Comparisons	T2	a	a
Nitrite	Dunn's Kruskal-Wallis Multiple Comparisons	T3	a	a
Oxalate	Dunn's Kruskal-Wallis Multiple Comparisons	T0	a	a
Oxalate	Dunn's Kruskal-Wallis Multiple Comparisons	T1	a	a
Oxalate	Dunn's Kruskal-Wallis Multiple Comparisons	T2	ab	ab
Oxalate	Dunn's Kruskal-Wallis Multiple Comparisons	T3	b	b
pH	Dunn's Kruskal-Wallis Multiple Comparisons	T0	b	b
pH	Dunn's Kruskal-Wallis Multiple Comparisons	T1	a	a
pH	Dunn's Kruskal-Wallis Multiple Comparisons	T2	a	a
pH	Dunn's Kruskal-Wallis Multiple Comparisons	T3	ab	ab
Phosphate	Dunn's Kruskal-Wallis Multiple Comparisons	T0	a	a
Phosphate	Dunn's Kruskal-Wallis Multiple Comparisons	T1	a	a
Phosphate	Dunn's Kruskal-Wallis Multiple Comparisons	T2	a	a
Phosphate	Dunn's Kruskal-Wallis Multiple Comparisons	T3	a	a
Potassium	Dunn's Kruskal-Wallis Multiple Comparisons	T0	b	b
Potassium	Dunn's Kruskal-Wallis Multiple Comparisons	T1	ab	ab
Potassium	Dunn's Kruskal-Wallis Multiple Comparisons	T2	ab	ab
Potassium	Dunn's Kruskal-Wallis Multiple Comparisons	T3	a	a
Pyruvate	Dunn's Kruskal-Wallis Multiple Comparisons	T0	a	a
Pyruvate	Dunn's Kruskal-Wallis Multiple Comparisons	T1	a	a
Pyruvate	Dunn's Kruskal-Wallis Multiple Comparisons	T2	a	a
Pyruvate	Dunn's Kruskal-Wallis Multiple Comparisons	T3	a	a
Sodium	Dunn's Kruskal-Wallis Multiple Comparisons	T0	a	a
Sodium	Dunn's Kruskal-Wallis Multiple Comparisons	T1	a	a

Sodium	Dunn's Kruskal-Wallis Multiple Comparisons	T2	b	b
Sodium	Dunn's Kruskal-Wallis Multiple Comparisons	T3	ab	ab
Succinate	Dunn's Kruskal-Wallis Multiple Comparisons	T0	a	a
Succinate	Dunn's Kruskal-Wallis Multiple Comparisons	T1	a	a
Succinate	Dunn's Kruskal-Wallis Multiple Comparisons	T2	a	a
Succinate	Dunn's Kruskal-Wallis Multiple Comparisons	T3	a	a
Sulfate	Dunn's Kruskal-Wallis Multiple Comparisons	T0	a	a
Sulfate	Dunn's Kruskal-Wallis Multiple Comparisons	T1	a	a
Sulfate	Dunn's Kruskal-Wallis Multiple Comparisons	T2	a	a
Sulfate	Dunn's Kruskal-Wallis Multiple Comparisons	T3	a	a
TC	Dunn's Kruskal-Wallis Multiple Comparisons	T0	a	a
TC	Dunn's Kruskal-Wallis Multiple Comparisons	T1	a	a
TC	Dunn's Kruskal-Wallis Multiple Comparisons	T2	a	a
TC	Dunn's Kruskal-Wallis Multiple Comparisons	T3	a	a
TN	Dunn's Kruskal-Wallis Multiple Comparisons	T0	a	a
TN	Dunn's Kruskal-Wallis Multiple Comparisons	T1	a	a
TN	Dunn's Kruskal-Wallis Multiple Comparisons	T2	a	a
TN	Dunn's Kruskal-Wallis Multiple Comparisons	T3	a	a
